# Metabolically healthy obese individuals present similar chronic inflammation level but less insulin-resistance than obese individuals with metabolic syndrome

**DOI:** 10.1371/journal.pone.0190528

**Published:** 2017-12-28

**Authors:** Andrea Elena Iglesias Molli, Alberto Penas Steinhardt, Ariel Pablo López, Claudio Daniel González, Jorge Vilariño, Gustavo Daniel Frechtel, Gloria Edith Cerrone

**Affiliations:** 1 CONICET-Universidad de Buenos Aires, Instituto de Inmunología, Genética y Metabolismo (INIGEM), Laboratorio de Diabetes y Metabolismo, Ciudad Autónoma de Buenos Aires, Buenos Aires, Argentina; 2 Universidad Nacional de Luján, Departamento de Ciencias Básicas, Laboratorio de Genómica Computacional, Luján, Buenos Aires, Argentina; 3 Fundación H.A. Barceló, Instituto Universitario de Ciencias de la Salud, Ciudad Autónoma de Buenos Aires, Buenos Aires, Argentina; 4 Universidad de Buenos Aires, Facultad de Medicina, Departamento de Farmacología, Cátedra II, Ciudad Autónoma de Buenos Aires, Buenos Aires, Argentina; 5 Hospital FLENI, Departamento de Cardiología, Ciudad Autonoma de Buenos Aires, Buenos Aires, Argentina; 6 Universidad de Buenos Aires, Facultad de Farmacia y Bioquímica, Departamento de Microbiología, Inmunología y Biotecnología, Cátedra de Genética, Ciudad Autónoma de Buenos Aires, Buenos Aires, Argentina; Universitat de les Illes Balears, SPAIN

## Abstract

The Metabolic Syndrome (MetS) is a cluster of cardiometabolic risk factors, usually accompanied by the presence of insulin resistance (IR) and a systemic subclinical inflammation state. Metabolically healthy obese (MHO) individuals seem to be protected against cardiometabolic complications. The aim of this work was to characterize phenotypically the low-grade inflammation and the IR in MHO individuals in comparison to obese individuals with MetS and control non obese. We studied two different populations: 940 individuals from the general population of Buenos Aires and 518 individuals from the general population of Venado Tuerto; grouped in three groups: metabolically healthy non-obese individuals (MHNO), MHO and obese individuals with MetS (MSO). Inflammation was measured by the levels of hs-CRP (high-sensitivity C reactive protein), and we found that MHO presented an increase in inflammation when compared with MHNO (Buenos Aires: p<0.001; Venado Tuerto: p<0.001), but they did not differ from MSO. To evaluate IR we analyzed the HOMA (Homoeostatic Model Assessment) values, and we found differences between MHO and MSO (Buenos Aires: p<0.001; Venado Tuerto: p<0.001), but not between MHNO and MHO. In conclusion, MHO group would be defined as a subgroup of obese individuals with an intermediate phenotype between MHNO and MSO individuals considering HOMA, hs-CRP and central obesity.

## Introduction

Metabolic Syndrome (MetS) is a cluster of interrelated cardiovascular and metabolic risk factors that predispose individuals to the development of aging-related diseases, such as type 2 diabetes mellitus (T2D) and cardiovascular disease (CVD). These well-known risk factors include abdominal obesity, hyperglycemia, hypertension and atherogenic dyslipidemia. From a physiopathological point of view, the cardiometabolic risk factors that characterize MetS are usually accompanied by the presence of insulin resistance (IR), and by a systemic subclinical inflammation state secondary to the hyperactivity of innate immunity [1].

The visceral adipose tissue is a dysfunctional tissue constituted by adipocytes and also by macrophages producing proinflammatory cytokines that contribute to the state of subclinical inflammation and to the development of IR, T2D and MetS. The behavior of this adipose tissue at visceral localization is different from that of subcutaneous adipose tissue, which presents a lower infiltration of macrophages and therefore a lower production of proinflammatory cytokines. Thus, visceral adipose tissue is a risk factor for T2D and CVD, whereas subcutaneous adipose tissue is not a risk factor for these pathologies [2, 3]. Subcutaneous adipose tissue also presents a different behavior considering its location in the abdomen (upper-body) or in the gluteofemoral region (lower-body). Lower-body adipose tissue demonstrates substantially lower rate of cytokine release, which results in fewer signs of inflammatory insult [4]. A higher percentage of lower-body adipose tissue was strongly associated with higher insulin sensitivity, and showed a lower risk of CVD [5]. Both circulating monocytes and macrophages deposited in adipose tissue produces proinflammatory cytokines, but are also able to produce anti-inflammatory molecules according to the metabolic status of the individual. The immune response that produces proinflammatory cytokines (characteristic of dysfunctional adipose tissue) or anti-inflammatory molecules has a dynamic behavior according to the metabolic state [6].

In the etiology of MetS, aging, genetics, inflammation, obesity and sedentary lifestyle are recognized as predisposing factors. Although abdominal obesity is the major risk factor of the MetS that predispose to the metabolic and cardiovascular diseases, there is a subgroup of obese individuals that seems to be protected against these obesity-related complications [7], known as obese individuals with uncomplicated obesity or metabolically healthy obese (MHO) [8]. The MHO phenotype, first described by Sims in 2001, includes individuals with an obese phenotype but without the presence of MetS and metabolic complications [9]. MHO individuals do not have a higher risk of CVD or T2D or an increased mortality than normal-weight individuals [10]. Some studies suggested that this population might have cardiovascular risk comparable to metabolically healthy non-obese individuals [11, 12]. The data from 13 studies suggested that individuals with metabolically healthy obesity are at higher risk of cardiovascular events than metabolically healthy normal weight (RR = 1.45; 95% CI RR = 1.20/1.70). However, the risk is considerably lower than metabolically unhealthy obese individuals [13]. It has been shown that MHO individuals have a higher risk of developing metabolic disorders than metabolically healthy individuals with normal weight. In a follow-up study, 33% of MHOs developed MetS after a mean time of 8.2 years. In this way, for some individuals, MHO phenotype could represent an initial state in the progression to metabolically unhealthy obesity [14]. Environmental and behavioral factors can modify healthy and unhealthy obesity sub-phenotypes and transitions from one to another sub-phenotype are possible. The MHO phenotype is characterized by preserved insulin sensitivity, relatively low visceral fat mass and less adipose tissue dysfunction [15]. It has also been shown that obese individuals who are insulin-sensitive have a significantly lower ectopic fat content in the liver than obese individuals who are IR [16].

Experimental and observational evidence suggests that inflammation may play a central role in the pathogenesis of cardiovascular disease [17]. High-sensitivity C reactive protein (hs-CRP) is associated with all parameters of the MetS and has been acknowledged to be an independent but not causal risk factor for incident CVD and to add prognostic value for CVD risk on top of the MetS criteria [18, 19].

Only few studies examine inflammation and IR in MHO. In this way there is still little evidence to suggest that other cardiometabolic risk markers, such as hs-CRP concentrations, could be used to better define metabolic health [13]. Wildman et al. in 2008 conducted a prevalence study in US adults, in which they proposed hs-CRP and HOMA as cardiometabolic abnormalities to define the state of metabolically healthy vs metabolically abnormal [20]. The aim of our work was to characterize phenotypically the low-grade inflammation by the evaluation of hs-CRP and its immediate consequence, the IR, in MHO individuals with respect to obese individuals with MetS (MSO) and metabolically healthy non-obese individuals (MHNO).

## Material and methods

For this work, two independent populations were recruited. From the general population of Buenos Aires, we recruit 940 individuals, who voluntary attended to the Hemotherapy Service of the Hospital de Clínicas “José de San Martín”, Buenos Aires. On the other hand, we randomly recruited 518 individuals following a stratified multistage sampling design, from the general population of Venado Tuerto, Province of Santa Fe. The prevalence of chronic metabolic diseases and the study of biochemical markers associated with CVD in the population of Venado Tuerto were already published [21]. The study was approved by the Ethics Committees of Hospital de Clínicas and Hospital de Venado Tuerto respectively, and all participants gave their written informed consent.

Individuals with body mass index (BMI) ≥ 30 kg/m2 were considered obese. In accordance with the ATPIII criteria [22], individuals with three or more of the following components were considered to have MetS: waist circumference ≥ 102 cm for men and ≥ 88 cm for women; systolic blood pressure (SBP) ≥ 130 mmHg, or diastolic blood pressure (DBP) ≥ 85 mmHg, or treated for hypertension; fasting plasma glucose (FPG) ≥ 100 mg/dl, or treated for T2D; high-density lipoprotein cholesterol (HDL-C) < 40 mg/dl for men and < 50 mg/dl for women, or treated for dyslipidemia; and triglycerides (TG) ≥ 150 mg/dl, or treated for dyslipidemia. We considered as ‘metabolically healthy’ those individuals who do not have MetS, but they may have two or less of the previous metabolic risk factors. MHO individuals were defined as obese individuals without MetS, in accordance with Alberti et al. [23] who published a consensus of several Clinical Societies. MHNO group consisted of individuals without obesity and MetS, and was considered a group with better metabolic condition than MHO. On the other hand, MSO group included obese individuals with MetS, and was considered a group with worse metabolic condition that MHO.

All the individuals attended with 12 hours of fasting to perform the anthropometric, clinical and biochemical determinations. Standardized protocols were used to determinate height, weight, waist circumference, SBP and DBP. BMI calculation was performed as weight (kg) / [height (m)]2. Serum was obtained by centrifugation of venous blood samples. FPG, HDL-C and TG were determined by enzymatic methods using commercial kits. Fasting serum insulin (FSI) was measured by electrochemiluminescence immunoassay with a commercial kit (Insulin, Roche Diagnostics, Mannheim, Germany) in a Cobas e411 (Roche Diagnostics, Mannheim, Germany), with a lower detection limit of 2 μU/mL. Homoeostatic Model Assessment (HOMA) was calculated as FPG (mM) * FSI (mIU/L) / 22.5. hs-CRP levels in serum were measured using particle enhanced immunonephelometry assay (Cardio Phase^®^ hs-CRP, Siemens) on a Siemens BN Prospect Nephelometer (Siemens Healthcare Diagnostics, Deerfield, IL, USA). In accordance with previous studies, hs-CRP values > 10 mg/l were considered to represent an acute inflammatory response [24]. All subjects completed a questionnaire detailing their self-reported physical activity and cigarette smoking habits. It was considered the physical activity level during leisure time and at work, and it was classified into two categories: sedentary lifestyle vs moderate to high physical activity. To measure the exposure to tobacco, we considered non-smokers, ex-smokers and active smokers.

Statistical analyses were performed using SPSS version 20.0 (IBM Corp., Armonk, NY, USA), and both populations were studied separately. The analysis of biochemical, clinical and anthropometric characteristics between groups was performed by multiple linear regression, and the study of dummy variables to compare MHO with the other two groups. We consider the groups MHNO, MHO and MSO as ordinal variable, since each group shows a worse metabolic condition than the previous one. In this analysis we use different covariates in order to eliminate their influence. We performed binary logistic regression to estimate the probability of having an increased HOMA and hs-CRP in function of the metabolic condition, age and gender. For this analysis we considered an increased HOMA when it takes values ≥ 2.5; and an increased hs-CRP when it takes values greater than or equal to the mean for each population. We also analyzed the probability that MHO individuals have an increased hs-CRP in function of the HOMA value, the number of components of MetS and each of the variables that determinate the diagnosis of MetS (waist circumference, SBP, DBP, FPG, TG and HDL-C), by binary logistic regression with age and gender as covariates. A p value below 0.05 was considered as statistically significant.

## Results

The Buenos Aires population consisted of 235 (25.0%) women and 705 (75.0%) men, with a mean age of 36.45 years (SD = 11.30; range: 18–65 years). Considering biochemical, clinical and anthropometric characteristics, we observed that 655 individuals of the cohort (69.7%) were MHNO, 121 (12.9%) were MHO and 164 (17.4%) were MSO. The Venado Tuerto population consisted of 343 (66.2%) women and 175 (33.8%) men, with a mean age of 45.71 years (SD = 15.19; range: 18–86 years). From this population, 282 individuals of the cohort (54.4%) were MHNO, 82 (15.8%) were MHO and 154 (29.7%) were MSO. Three major issues must be considered: firstly, the initial aim of the recruitment was an epidemiological study of a general population afterward subdivided in the clinical groups, it was not made for MHO study in particular. Second, the prevalence of MHO depends on the definition criteria used and it differs through the different publications. Third, there were differences in the male and female percentages between both populations which may explain the frequencies of MHNO, MHO and MSO observed, so gender correction was made in the statistical analysis.

In Tables 1 and 2 it can be seen the anthropometric, clinical and biochemical characteristics of the populations. Significant differences in age were observed when comparing the three groups in both populations, therefore all subsequent statistical analysis was performed with age corrections as well. We found differences in height only between MHNO and MHO groups in Venado Tuerto population. Using age, gender, height, physiscal activity and tobaco exposure as covariates, we found statistically significant regressions in all the other variables analyzed between the three groups. The comparison between the MHO and MHNO groups, which differ by the presence of obesity, showed differences in BMI and waist circumference. For the Venado Tuerto population, SBP, DBP and HDL-C showed differences as well. We found no differences in FPG and TG between MHO and MHNO in neither of the two populations. When we compared MHO with MSO groups, which are both groups of obese individuals but they differ by the presence of MetS, we found differences in SBP, DBP, FPG, HDL-C and TG, as expected. In Venado Tuerto population we also found significantly differences in BMI and waist circumference.

**Table 1:** Anthropometric, clinical and biochemical characteristics of the Buenos Aires population grouped according to the metabolic condition.

**A**						
	**Groups**			**Statistical analysis**		
	**MHNO**	**MHO**	**MSO**			
	M ± SD	M ± SD	M ± SD	P	r	95%CI r
Age (years)*	34 ± 11	39 ± 12	43 ± 10	< 0.001	4.63	3.74 / 5.51
Males (%)	72.8	71.9	86.0			
Females (%)	27.2	28.1	14.0			
Height (cm)#	170 ± 9	168 ± 9	170 ± 9	NS		
BMI (kg/m^2^)ᵗ	25.16 ± 2.49	33.61 ± 3.82	34.40 ± 3.86	< 0.001	4.82	4.52 / 5.11
Waist circumference (cm)ᵗ	87 ± 9	107 ± 9	111 ± 9	< 0.001	11.20	10.43 / 11.97
SBP (mmHg)ᵗ	121 ± 10	122 ± 11	133 ± 10	< 0.001	4.45	3.56 / 5.34
DBP (mmHg)ᵗ	77 ± 8	79 ± 7	83 ± 7	< 0.001	2.37	1.72 / 3.02
FPG (mg/dl)ᵗ	88 ± 9	90 ± 9	99 ± 16	< 0.001	3.77	2.80 / 4.73
HDL-C (mg/dl)ᵗ	46 ± 12	45 ± 10	36 ± 8	< 0.001	-4.78	-5.74 / -3.83
TG (mg/dl)ᵗ	99 ± 54	112 ± 70	194 ± 99	< 0.001	36.84	30.81 / 42.84
HOMAᵗ	2.81 ± 1.55	3.83 ± 1.93	5.68 ± 3.05	< 0.001	1.35	1.17 / 1.53
hs-CRP (mg/l)ᵗ	1.48 ± 1.44	2.53 ± 2.11	2.76 ± 2.26	< 0.001	0.64	0.47 / 0.81
**B**						
	**Test *post-hoc***					
	**MHNO vs MHO**			**MHO vs MSO**		
	p	r	95%CI r	p	r	95%CI r
Age (years)*	< 0.001	4.95	2.86 / 7.03	0.001	4.22	1.70 / 6.75
Height (cm)#	NS			NS		
BMI (kg/m^2^)ᵗ	< 0.001	8.16	7.55 / 8.76	NS		
Waist circumference (cm)ᵗ	< 0.001	18.97	17.34 / 20.59	NS		
SBP (mmHg)ᵗ	NS			< 0.001	9.59	7.21 / 11.97
DBP (mmHg)ᵗ	NS			< 0.001	3.61	1.84 / 5.37
FPG (mg/dl)ᵗ	NS			< 0.001	7.67	5.08 / 10.27
HDL-C (mg/dl)ᵗ	NS			< 0.001	-8.39	-10.97 / -5.80
TG (mg/dl)ᵗ	NS			< 0.001	71.60	55.47 / 87.73
HOMAᵗ	< 0.001	0.93	0.51 / 1.34	< 0.001	1.88	1.38 / 2.38
hs-CRP (mg/l)ᵗ	< 0.001	1.04	0.65 / 1.43	NS		

*Multiple linear regression and dummy variables analysis.

^#^Multiple linear regression and dummy variables analysis (covariates: age and gender).

ᵗMultiple linear regression and dummy variables analysis (covariates: age, gender, height, physical activity and tobacco exposure).

MHNO: Metabolically Healthy Non-obese individuals; MHO: Metabolically Healthy Obese individuals; MSO: Obese individual with Metabolic Syndrome; M: mean; SD: standard deviation; r: regression coefficient; 95%CI r: 95% confidence interval for the regression coefficient; BMI: Body mass index; SBP: systolic blood pressure; DBP: diastolic blood pressure; FPG: fasting plasma glucose; HDL-C: high-density lipoprotein cholesterol; TG: triglycerides; HOMA: Homoeostatic Model Assessment; hs-CRP: High-Sensitive C reactive protein; NS: not significant.

**Table 2:** Anthropometric, clinical and biochemical characteristics of the Venado Tuerto population grouped according to the metabolic condition.

**A**						
	**Groups**			**Statistical analysis**		
	**MHNO**	**MHO**	**MSO**			
	M ± SD	M ± SD	M ± SD	P	r	95%CI r
Age (years)*	42 ± 15	46 ± 15	51 ± 13	< 0.001	4.44	3.00 / 5.87
Males (%)	33.0	37.8	33.1			
Females (%)	67.0	62.2	66.9			
Height (cm)#	164 ± 9	162 ± 12	163 ± 10	NS		
BMI (kg/m^2^)ᵗ	24.55 ± 2.79	34.35 ± 5.39	36.78 ± 5.74	< 0.001	6.23	5.77 / 6.68
Waist circumference (cm)ᵗ	86 ± 9	107 ± 10	114 ± 12	< 0.001	13.81	12.77 / 14.86
SBP (mmHg)ᵗ	115 ± 19	125 ± 19	139 ± 22	< 0.001	8.57	6.84 / 10.31
DBP (mmHg)ᵗ	70 ± 12	78 ± 14	85 ± 13	< 0.001	5.81	4.66 / 6.96
FPG (mg/dl)ᵗ	91 ± 21	91 ± 8	114 ± 42	< 0.001	10.11	7.19 / 13.02
HDL-C (mg/dl)ᵗ	55 ± 13	52 ± 11	45 ± 11	< 0.001	-5.88	-7.08 / -4.68
TG (mg/dl)ᵗ	98 ± 48	99 ± 38	173 ± 99	< 0.001	35.03	28.15 / 41.91
HOMAᵗ	2.00 ± 1.53	3.21 ± 2.64	6.10 ± 7.83	< 0.001	1.93	1.57 / 2.28
hs-CRP (mg/l)ᵗ	1.99 ± 1.95	3.09 ± 2.56	3.68 ± 2.53	< 0.001	0.90	0.65 / 1.15
**B**						
	**Test *post-hoc***					
	**MHNO vs MHO**			**MHO vs MSO**		
	p	r	95%CI r	p	r	95%CI r
Age (years)*	0.027	4.09	0.46 / 7.71	0.017	4.83	0.88 / 8.77
Height (cm)#	0.042	1.94	0.08 / 3.81	NS		
BMI (kg/m^2^)ᵗ	< 0.001	9.51	8.45 / 10.57	< 0.001	2.61	1.46 / 3.76
Waist circumference (cm)ᵗ	< 0.001	21.12	18.70 / 23.53	< 0.001	5.74	3.11 / 8.37
SBP (mmHg)ᵗ	0.005	5.98	1.78 / 10.18	< 0.001	11.43	6.87 / 16.00
DBP (mmHg)ᵗ	< 0.001	5.94	3.14 / 8.74	< 0.001	5.67	2.63 / 8.70
FPG (mg/dl)ᵗ	NS			< 0.001	22.47	14.81 / 30.13
HDL-C (mg/dl)ᵗ	0.021	-3.44	-6.37 / -0.51	< 0.001	-8.57	-11.75 / -5.40
TG (mg/dl)ᵗ	NS			< 0.001	71.84	53.90 / 89.78
HOMAᵗ	0.002	1.39	0.54 / 2.25	< 0.001	2.51	1.58 / 3.44
hs-CRP (mg/l)ᵗ	< 0.001	1.10	0.49 / 1.72	NS		

*Multiple linear regression and dummy variables analysis.

^#^Multiple linear regression and dummy variables analysis (covariates: age and gender).

ᵗMultiple linear regression and dummy variables analysis (covariates: age, gender, height, physical activity and tobacco exposure).

MHNO: Metabolically Healthy Non-obese individuals; MHO: Metabolically Healthy Obese individuals; MSO: Obese individual with Metabolic Syndrome; M: mean; SD: standard deviation; r: regression coefficient; 95%CI r: 95% confidence interval for the regression coefficient; BMI: Body mass index; SBP: systolic blood pressure; DBP: diastolic blood pressure; FPG: fasting plasma glucose; HDL-C: high-density lipoprotein cholesterol; TG: triglycerides; HOMA: Homoeostatic Model Assessment; hs-CRP: High-Sensitive C reactive protein; NS: not significant.

To evaluate the IR in these groups we analyzed the HOMA values, and we found that in both populations, as the metabolic condition worsens, the median HOMA increase with significant differences between every pair of groups (Tables 1 and 2). When we performed the same analysis using waist circumference as a covariate, we found differences in HOMA between MHO and MSO (Buenos Aires: p<0.001; r = 1.82; 95% CI r = 1.33/2.31; Venado Tuerto: p<0.001; r = 2.10; 95% CI r = 1.17/3.03). Since we found no differences between MHNO and MHO groups, we considered that IR was associated with waist circumference, that represents abdominal obesity, but not with obesity itself. We also found an increased risk to have HOMA´s value ≥ 2.5 as metabolic condition worsens in both populations (Buenos Aires: p<0.001; OR = 3.227; 95% CI OR = 2.476/4.205; Venado Tuerto: p<0.001; OR = 3.691; 95% CI OR = 2.871/4.744).

In Tables 1 and 2 it can be seen that the level of inflammation, measured by the hs-CRP, increases as the metabolic condition worsens; where MHO individuals presented increased inflammation when compared to MHNO, but they did not differ from MSO in both populations. The probability to have an increased value of hs-CPR (values greater than or equal to the mean for each population: Buenos Aires ≥ 1.85 mg/l; Venado Tuerto ≥ 2.71 mg/l) was significantly associated to the metabolic condition (Buenos Aires: p<0.001; OR = 1.903; 95% CI OR = 1.530/2.366; Venado Tuerto: p<0.001; OR = 2.095; 95% CI OR = 1.647/2.665). In the MHO individuals, the only variable that was associated with a higher risk of having an increased value of hs-CPR was the waist circumference (Buenos Aires: p = 0.006; OR = 1.085; 95% CI OR = 1.023/1.151; Venado Tuerto: p = 0.040; OR = 1.074; 95% CI OR = 1.003/1.150), even though with a low risk. We did not associate the HOMA value, the number of components of MetS and the other variables that determinate the diagnosis of MetS (SBP and DBP, FPG, TG and HDL-C) with an increased value of hs-CPR in MHO individuals.

## Discussion

Mechanisms that could explain the favorable metabolic profile of MHO individuals are not well understood. The aim of this work was to characterize phenotypically the low-grade inflammation and its immediate consequence, the IR, in MHO individuals with respect to obese individuals with MetS and control individuals without obesity and MetS. The main finding in the present work was the presence of a similar degree of inflammation, measured by hs-CRP levels, when we compare MHO with MSO, which differs significantly to MHNO. The immediate consequence of the inflammation, the IR, evaluated by HOMA as a surrogate, did not differ between MHO and MHNO, but it was different between MHO and MSO, when we used waist circumference as a covariate. This demonstrates that the development of IR is closely related to central obesity in MHO, and related to the other metabolic variables in MSO. We could replicate these findings in two different populations of our country.

Philips et al. found that the level of hs-CRP was similar between MHO and MSO in accordance to our results [25]. Martinez Larrad et al. found similar results to our work when comparing MHO and MSO, in a Spanish population genetically related to ours. They found similar levels of hs-CRP with higher level of HOMA in MSO than in MHO, although no statistically significant differences were observed [26]. Finally, similar levels of hs-CRP between MSO and MHO were recently described in a large sample of Brazilian population as well [27].

Shi et al. demonstrated the link between metabolism and the innate immune response, and how inflammation is involved in processes that constitute the metabolic complications of obesity, such as IR [28]. We postulate that MHO individuals are protected from the metabolic complications of inflammation such as IR and CVD. A recent review demonstrated that the MHO phenotype was not linked with adverse CVD outcomes; however, systemic inflammation was not explored in this review [29]. This is not the case of MSO which presented the same elevated levels of hs-CRP than MHO but higher levels of IR and developed greater metabolic complications. In this way, chronic low-grade inflammation plays a role in the pathogenesis of IR in MSO, with elevated circulating levels of hs-CRP and proinflammatory cytokines associated with increased cardiometabolic risk [30, 31].

Wang et al. generated Brd2-knockout mice. Brd2 binds not only activators like E2F proteins, Hats and Hdacs (histone deacetylases) to regulate the expression of diverse genes, Brd2 normally function is to inhibit adipogenic differentiation and is linked to insulin-resistance and cancer. The Brd2 knockout mice are heterozygous and produce obese mice metabolically healthy. In this way, similar to our MHO patients, this animal model presents insulin-sensibility and increased serum levels of pro-inflammatory cytokines, such as TNF-α and IL-1β. The Brd2 knockout mice are a good animal model to replicate the metabolically health obese state [32].

In both populations we saw that the groups with a worse metabolic condition had a higher risk of having increased hs-CRP and HOMA index. The different genetic architecture between MSO and MHO could explain the results found in our work, in this way recently a Genome Wide Association Study (GWAS) have been published which could determine the different traits related to obesity (adiposity, abdominal adiposity, BMI, waist to hip ratio) according to different SNPs that characterize each of them. Some of those SNPs and principally Genetics Risk Scores (GRS) constructed with them have been correlated with hs-CRP level and other proinflammatory molecules such as interleukin 6 [33]. Interestingly, van Wijk et al. found that hs-CRP increases the risk of CVD for MHO participants with hs-CRP ≥ 2 mg/L than metabolically healthy participants with hs-CRP < 2 mg/L (p = 0.066; HR = 1.59; 95% CI HR = 0.97/2.62), although this trend did not reach statistical significance. hs-CRP appears to be an easy and widely available molecule for identifying a low-risk subpopulation among metabolically healthy obese persons [34].

Actually, it is accepted that visceral and ectopic fat content is associated with a decrease in insulin sensitivity. In that sense, although obese individuals have larger quantities of fat mass with respect to non-obese individuals, we observed that MHO individuals present similar levels of insulin sensitivity than MHNO individuals, when the statistical analysis is corrected by waist circumference. This could be explained by the reported 49% lesser visceral adipose tissue observed in MHO individuals (as measured from computed tomography) in comparison to obese subjects with MetS [7]. Also, when we evaluated the IR, we found that MHO had significantly lower HOMA index compared to MSO patients and similar results have been published for metabolically healthy peripherally obese [22].

The main strength of our work is that we were able to replicate the results in two independent populations of Argentina. Our work has some limitations: i) there are different criteria to define MHO individuals and their prevalence depends on the definition criteria used; ii) we cannot establish cause-effect relationships, since our study follows a cross-sectional design.

In conclusion, MHO group would be defined as a subgroup of obese individuals with an intermediate phenotype between MHNO and MSO individuals considering HOMA; hs-CRP and central obesity. We must recognize that the MHO phenotype presents a proinflammatory state, that raise questions about the physiopathological mechanism and its potential clinical implications. A better understanding of the physiopathological aspects of these metabolic diseases will help in the identification of individuals with high risk to develop these entities, as well as the identification of groups of individuals with common phenotypic characteristics, facilitating their prevention, delay or protection by individualized treatments.
